# Evaluation of CNV detection tools for NGS panel data in genetic diagnostics

**DOI:** 10.1038/s41431-020-0675-z

**Published:** 2020-06-19

**Authors:** José Marcos Moreno-Cabrera, Jesús del Valle, Elisabeth Castellanos, Lidia Feliubadaló, Marta Pineda, Joan Brunet, Eduard Serra, Gabriel Capellà, Conxi Lázaro, Bernat Gel

**Affiliations:** 1 Hereditary Cancer Group, Program for Predictive and Personalized Medicine of Cancer, Germans Trias i Pujol Research Institute (PMPPC-IGTP), Campus Can Ruti, Badalona, Spain; 2 Hereditary Cancer Program, Joint Program on Hereditary Cancer, Catalan Institute of Oncology, Institut d’Investigació Biomèdica de Bellvitge—IDIBELL, L’Hospitalet de Llobregat, Barcelona, Spain; 3 Centro de Investigación Biomédica en Red Cáncer (CIBERONC), Instituto de Salud Carlos III, Madrid, Spain; 4 Hereditary Cancer Program, Catalan Institute of Oncology, IDIBGi, Girona, Spain

**Keywords:** Genetic testing, Data processing

## Abstract

Although germline copy-number variants (CNVs) are the genetic cause of multiple hereditary diseases, detecting them from targeted next-generation sequencing data (NGS) remains a challenge. Existing tools perform well for large CNVs but struggle with single and multi-exon alterations. The aim of this work is to evaluate CNV calling tools working on gene panel NGS data and their suitability as a screening step before orthogonal confirmation in genetic diagnostics strategies. Five tools (DECoN, CoNVaDING, panelcn.MOPS, ExomeDepth, and CODEX2) were tested against four genetic diagnostics datasets (two in-house and two external) for a total of 495 samples with 231 single and multi-exon validated CNVs. The evaluation was performed using the default and sensitivity-optimized parameters. Results showed that most tools were highly sensitive and specific, but the performance was dataset dependant. When evaluating them in our diagnostics scenario, DECoN and panelcn.MOPS detected all CNVs with the exception of one mosaic CNV missed by DECoN. However, DECoN outperformed panelcn.MOPS specificity achieving values greater than 0.90 when using the optimized parameters. In our in-house datasets, DECoN and panelcn.MOPS showed the highest performance for CNV screening before orthogonal confirmation. Benchmarking and optimization code is freely available at https://github.com/TranslationalBioinformaticsIGTP/CNVbenchmarkeR.

## Introduction

Next-generation sequencing (NGS) is an outstanding technology to detect single-nucleotide variants and small deletion and insertion variants in genetic testing for Mendelian conditions. However, detection of large rearrangements such as copy-number variants (CNV) from NGS data is still challenging due to issues intrinsic to the technology including short read lengths and GC-content bias [1]. Nevertheless, it is well recognized that germline CNVs are the genetic cause of several hereditary diseases [2], so their analysis is a necessary step in a comprehensive genetic diagnostics strategy.

The gold standards for CNV detection in genetic diagnostics are multiplex ligation-dependent probe amplification (MLPA) and array comparative genomic hybridization (aCGH) [3, 4]. Both methods are time consuming and costly, so frequently only a subset of genes is tested, excluding others from the analysis, especially when using single-gene approaches. Therefore, the possibility of using NGS data as a first CNV screening step would decrease the number of MLPA/aCGH tests required and would free up resources.

Many tools for CNVs detection from NGS data have been developed [5–7]. Most of them can reliably call large CNVs (in the order of megabases) but show poor performance when dealing with small CNVs affecting only one or a few small exons, which are CNVs frequently involved in several genetic diseases [8]. In addition, most of these tools were designed to work with whole-genome or whole-exome data and struggle with the sparser data from NGS gene panels used in routine genetic testing. Therefore, the challenge is to identify a tool able to detect CNVs from NGS panel data at a single-exon resolution with sufficient sensitivity to be used as a screening step in a diagnostic setting.

**Fig. 1:**
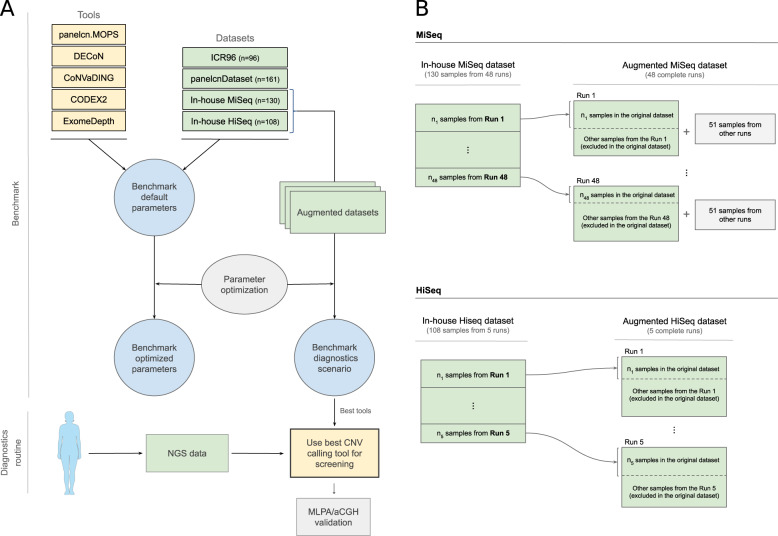
Benchmark design and augmented datasets. **a** The panel shows the benchmark design and the objective of applying the results in the diagnostics routine. **b** To evaluate the diagnostics scenario, a new dataset was built for each run belonging to the original dataset. The augmented datasets contained all the samples originally sequenced in the run and, in the case of the MiSeq datasets (upper), a set of 51 samples with no known CNV from different runs (MLPA multiplex ligation-dependent probe amplification; aCGH array comparative genomic hybridization; NGS next-generation sequencing; CNV copy-number variant).

Other benchmarks of CNV calling tools on targeted NGS panel data have been published. However, they were performed by the authors of the tools and executed against a single dataset [9–13], or used mainly simulated data with a small number of validated CNVs [14]. The aim of this work is to perform an independent benchmark of multiple CNV calling tools, optimizing, and evaluating them against multiple datasets generated in diagnostics settings, to identify the most suitable tools to be used for genetic diagnostics (Fig. 1).

## Materials and methods

### Datasets and tools

Four datasets were included in this benchmark (ICR96 exon CNV validation series [15], panelcnDataset [11], In-house MiSeq and In-House HiSeq) (Table 1) with data from two hybridization-based target capture NGS panels designed for hereditary cancer diagnostics: TruSight Cancer Panel (Illumina, San Diego, CA, USA) and I2HCP [16]. All datasets were generated in real diagnostics settings and contained single and multi-exon CNVs, all of them validated by MLPA. Negative MLPA data, meaning no detection of any CNV, were also available for a subset of genes. Detailed information on MLPA-detected CNVs for each dataset can be found in Supplementary files 2–5.

**Table 1:** Datasets used in the benchmark.

	Samples	Validated genes with CNV	Single-exon CNVs	Multi-exon CNVs	Deletion CNVs	Duplication CNVs	Validated genes with no CNV	Sequencing	Availability	Additional information
ICR96	96	68	25	43	51	17	1752 (96.3% of total)	TruSight Cancer Panel v2 (100 genes), HiSeq, 2 × 101 bp reads	European genome-phenome Archive EGAD00001003335	Samples obtained from one run
panelcnDataset	161	41	13	28	36	5	416 (91% of total)	TruSight Cancer Panel (94 genes), MiSeq, 2 × 151 bp reads	European Genome-phenome Archive EGAS00001002481	Only 161 of 170 samples were used. See Supplementary file 1
In-house MiSeq	130	64	19	45	56	8	167 (72.3% of total)	I2HCP Panel v2.0–v2.2 (122–135 genes), MiSeq, 2 × 300 bp reads	European Genome-phenome Archive EGAS00001004316	Samples obtained from 48 runs. Three samples had a CNV in mosaicism
In-house HiSeq	108	58	18	40	49	9	176 (75.2% of total)	I2HCP panel v2.0–v2.2 (122–135 genes), HiSeq, 2 × 251 bp reads	European Genome-phenome Archive EGAS00001004316	Samples obtained from 5 runs. Two samples had CNV in mosaicism

Samples from the In-house MiSeq and in-house HiSeq datasets were generated at the ICO-IGTP Joint Program for Hereditary Cancer and are available at the EGA under the accession number EGAS00001004316. In addition to these samples, a total of 1103 additional samples (505 MiSeq and 598 HiSeq), with no CNVs detected in the subset of genes tested by MLPA, were used to build the augmented datasets used in the diagnostics scenario analysis. Informed consent was obtained for all samples in the in-house datasets.

Five tools were tested in the benchmark (Table 2): CoNVaDING v1.2.0 [9], DECoN v1.0.1 [10], panelcn.MOPS v1.0.0 [11], ExomeDepth v1.1.10 [17], and CODEX2 v1.2.0 [18].

**Table 2:** Tools tested in the benchmark.

	Language	Version	Number of parameters used in the benchmark	Reports no calls	Availability	Methods
CODEX2	R package	1.2.0^a^	10	No	https://github.com/yuchaojiang/CODEX2	Based on CODEX package, it models the GC content bias and normalizes the read depth data for CNV detection via a Poisson latent factor model.
CoNVaDING	Perl program	1.2.0	7	Yes	https://github.com/molgenis/CoNVaDING	Combination of ratio scores and Z-scores of the sample of interest compared to the selected normalized control samples.
DECoN	R program	1.0.1	3	Yes	https://github.com/RahmanTeam/DECoN	Modifies ExomeDepth package by altering the hidden Markov model probabilities to depend upon the distance between exons.
ExomeDepth	R package	1.1.10	4	No	https://github.com/vplagnol/ExomeDepth	Beta-binomial model with GC correction and hidden Markov model to combine likelihood across exons.
panelcn.MOPS	R package	1.0.0	13	Yes	https://github.com/bioinf-jku/panelcn.mops	Adaptation of cn.MOPS package, which decomposes variations in coverage across samples into integer copy numbers and noise by means of its mixture components and Poisson distributions.

^a^CODEX2 script for panel setting (Codex2_targeted.R) was obtained from version dated at on Sep 12, 2017.

### Data preprocessing

All samples were aligned to the GRCh37 human genome assembly using BWA mem v0.7.12 [19, 20]. SAMtools v0.1.19 [21] was used to sort and index BAM files. No additional processing or filtering was applied to the BAM files.

### Regions of interest

The regions of interest (ROIs) were dependent on the dataset. For TruSight based datasets, ICR96 and panelcnDataset, we used the targets bed file published elsewhere [10] with some modifications: the fourth column was removed, the gene was added and it was sorted by chromosome and start position (Supplementary file 6). For in-house datasets, we generated a target bed file containing all coding exons from all protein-coding transcripts of genes in the I2HCP panel v2.1 (Supplementary file 7). These data were retrieved from Ensembl BioMart version 67 [22] (http://may2012.archive.ensembl.org). All genes tested by MLPA and used in the benchmark were common to all I2HCP versions (v2.0-2.2).

### Benchmark evaluation metrics

The performance of each tool for CNVs detection was evaluated at two levels: per ROI and per gene.

Per ROI metrics treated all ROI as independent entities, assigning each of them a correctness value: true positive (TP) or true negative (TN) if the tool matched the results of MLPA, false negative (FN) if the tool missed a CNV detected by MLPA and false positive (FP) if the tool called a CNV not detected by MLPA. This is the most fine-grained metric.

Per gene metrics consider the fact that most MLPA kits cover a whole gene and so the true CNVs would be detected by MLPA when confirming any CNV call in any ROI of the affected gene. Therefore, per gene metrics assigned a correctness value to each gene taking into account all its exons: TP if one of its ROIs was a TP; FN if MLPA detected a CNV in at least one of its ROIs and none of them were detected by the tool; FP if the tool called a CNV in at least one ROI and none of them were detected by MLPA; TN if neither MLPA nor the tool detected a CNV in any of its ROIs.

For each tool against each dataset and evaluation level various performance metrics were computed: sensitivity defined as TP/(TP + FN), specificity defined as TN/(TN + FP), positive predictive value (PPV) defined as TP/(TP + FP), negative predictive value (NPV) defined as TN/(TN + FN), false negative rate (FNR) defined as FN/(FN + TP), false positive rate (FPR) defined as FP/(FP + TN), and F1 score (F1) defined as 2TP/(2TP + FP + FN).

### Parameter optimization

Parameters of each tool were optimized against each dataset to maximize sensitivity while limiting specificity loss: each dataset was split into two halves, a training set used to optimize tool parameters and a validation set to evaluate them (Supplementary file 8). The optimization algorithm followed a greedy approach: a local optimization was performed at each step with the aim of obtaining a solution close enough to the global optimum. Further details of the optimization algorithm can be found in Supplementary file 9.

### Benchmarking framework execution

An R framework, CNVbenchmarkeR, was built to perform the benchmark in an automatically and configurable way. Code and documentation are available at https://github.com/TranslationalBioinformaticsIGTP/CNVbenchmarkeR. Each selected tool was first executed against each dataset using default parameters as defined in tool documentation and then using the optimized parameters. Default and optimized parameter values can be found in Supplementary file 10. Tool outputs were processed with R v3.4.2, Bioconductor v3.5 [23], plyr [24], GenomicRanges [25], and biomaRt [26]. Plots were created with ggplot2 [27]. Confidence intervals (CIs) were calculated with epiR v1.0-14 at a CI of 95%. In addition, for each dataset, all executions were repeated to compare performance on two subsets: one excluding single-exon CNVs samples and one excluding multi-exon CNVs samples.

### Diagnostics scenario evaluation

The In-house MiSeq and In-house HiSeq datasets were composed of a selection of samples from different sequencing runs. In a real diagnostics scenario, the objective is to analyze a new run with all its sequenced samples. To simulate and evaluate the diagnostics scenario, we built the augmented datasets (Fig. 1), which contained all the samples from the sequencing runs instead of a selection of them. For the augmented datasets, the tools were executed against each run and metrics were computed by combining the results of all runs. Since some tools recommend more than 16 samples for optimal performance, we added 51 samples from other runs with no known CNVs when executing the tools on the runs of the augmented MiSeq dataset.

We also defined a new metric, whole diagnostics strategy, to take into account that in a diagnostics setting all regions where the screening tool was not able to produce a result (no call) should be identified and tested by other methods. Thus, any gene containing at least one positive call or no call in a ROI was considered as a positive call of the whole gene: TP if the gene contained at least one ROI affected by a CNV; FP if the gene did not contain any ROI affected by a CNV. In addition, if a tool identified a ROI both as a deletion and a duplication, it was considered a no call when computing metrics.

## Results

To identify the CNV calling tools that could be used as a screening step in a genetic diagnostics setting, we needed first to select the candidate tools, and then to evaluate their performance with a special emphasis on the sensitivity, both with their default parameters and with dataset-dependent optimized parameters.

### CNV calling tool selection

The first in the benchmark was to identify candidate tools that have shown promising results. After a literature search process, we selected five CNV calling tools to be evaluated (Table 2), all of them based on depth-of-coverage analysis. Three tools have been reported to perform well on NGS panel data at single-exon resolution: CoNVaDING v1.2.0 [9], DECoN v1.0.1 [10], and panelcn.MOPS v1.0.0 [11]. ExomeDepth v1.1.10 [17] was included due to its high performance in benchmarks on WES data [28, 29] and because the developers reported good performance with panel data (https://github.com/vplagnol/ExomeDepth). CODEX2 v1.2.0 was included due to the high sensitivity shown on WES data [18] and the availability of specific scripts for panel data (https://github.com/yuchaojiang/CODEX2).

### Benchmark with default parameters

We executed each tool on each dataset with the default parameters and computed evaluation statistics at two levels: per ROI and per gene (see “Methods”).

**Fig. 2:**
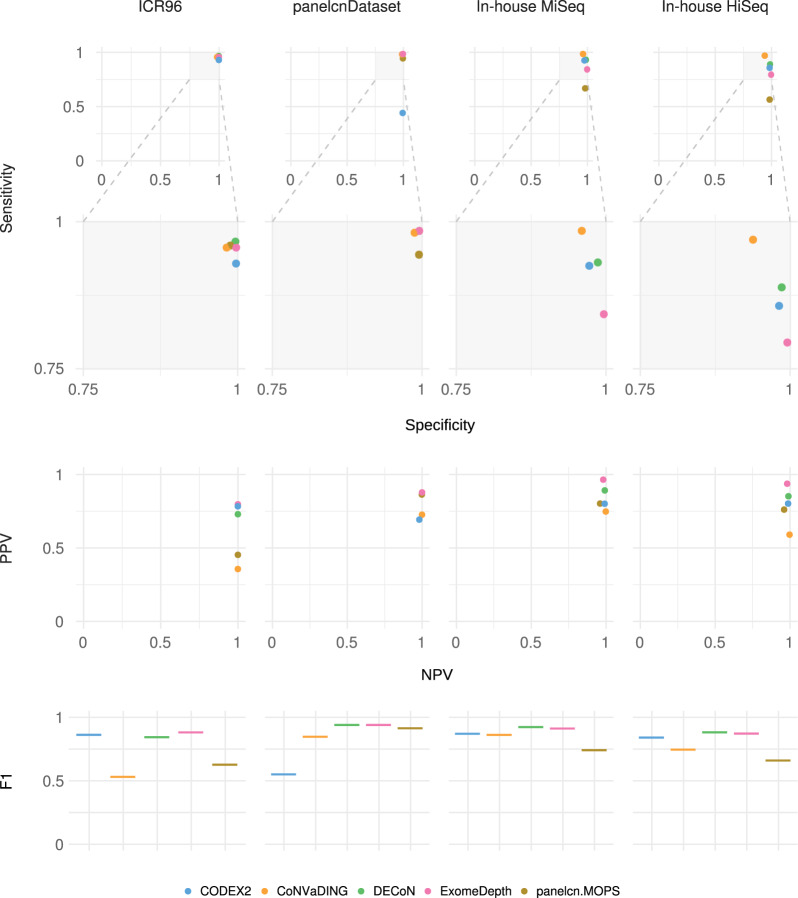
Benchmark results with default parameters: per ROI metrics. Shows results when executing tools with the default parameters and computing the per ROI metrics. ExomeDepth and DECoN tools obtained same sensitivity and specificity in panelcnDataset (ROI region of interest; PPV positive predictive value; F1 F1 score).

Regarding the per ROI metric, most tools showed sensitivity and specificity values over 0.75, with sensitivity in general over 0.9 (Fig. 2 and Table 3). However, tool performance varied across datasets. For the ICR96 and panelcnDataset datasets, specificity was always higher than 0.98, while sensitivity remained higher than 0.94 (with the exception of CODEX2). This performance was not achieved when using the in-house datasets, where lower sensitivity and specificity can be observed, and only CoNVaDING obtained sensitivity close to 1 at the expense of a lower specificity.

**Table 3:** Bechmark results with default parameters and per ROI metrics.

Dataset	Tool	TP	TN	FP	FN	Total	Sensitivity	Specificity	PPV	NPV	F1	FNR	FPR
ICR96	DECoN	286	28473	106	10	28875	0.9662	0.9963	0.7296	0.9996	0.8314	0.0338	0.0037
	panelcn.MOPS	284	28236	343	12	28875	0.9595	0.988	0.453	0.9996	0.6154	0.0405	0.0120
	CoNVaDING	283	28068	511	13	28875	0.9561	0.9821	0.3564	0.9995	0.5193	0.0439	0.0179
	exomedepth	283	28507	72	13	28875	0.9561	0.9975	0.7972	0.9995	0.8694	0.0439	0.0025
	CODEX2	275	28503	76	21	28875	0.9291	0.9973	0.7835	0.9993	0.8501	0.0709	0.0027
panelcnDataset	DECoN	317	9442	44	5	9808	0.9845	0.9954	0.8781	0.9995	0.9283	0.0155	0.0046
	panelcn.MOPS	304	9438	48	18	9808	0.9441	0.9949	0.8636	0.9981	0.9021	0.0559	0.0051
	CoNVaDING	316	9367	119	6	9808	0.9814	0.9875	0.7264	0.9994	0.8349	0.0186	0.0125
	exomedepth	317	9442	44	5	9808	0.9845	0.9954	0.8781	0.9995	0.9283	0.0155	0.0046
	CODEX2	142	9423	63	180	9808	0.441	0.9934	0.6927	0.9813	0.5389	0.5590	0.0066
In-house MiSeq	DECoN	486	4189	59	36	4770	0.931	0.9861	0.8917	0.9915	0.911	0.0690	0.0139
	panelcn.MOPS	349	4162	86	173	4770	0.6686	0.9798	0.8023	0.9601	0.7294	0.3314	0.0202
	CoNVaDING	513	4076	173	8	4770	0.9846	0.9593	0.7478	0.998	0.85	0.0154	0.0407
	exomedepth	440	4232	16	82	4770	0.8429	0.9962	0.9649	0.981	0.8998	0.1571	0.0038
	CODEX2	483	4128	120	39	4770	0.9253	0.9718	0.801	0.9906	0.8587	0.0747	0.0282
In-house HiSeq	DECoN	351	4197	61	44	4653	0.8886	0.9857	0.8519	0.9896	0.8699	0.1114	0.0143
	panelcn.MOPS	223	4188	70	172	4653	0.5646	0.9836	0.7611	0.9606	0.6483	0.4354	0.0164
	CoNVaDING	382	3994	265	12	4653	0.9695	0.9378	0.5904	0.997	0.7339	0.0305	0.0622
	exomedepth	314	4237	21	81	4653	0.7949	0.9951	0.9373	0.9812	0.8603	0.2051	0.0049
	CODEX2	324	4195	80	54	4653	0.8571	0.9813	0.802	0.9873	0.8286	0.1429	0.0187

*TP* true positive, *TN* true negative, *FP* false positive, *FN* false negative, *PPV* positive predictive value, *NPV* negative predictive value, *F1* F1 score, *FNR* false negative rate, *FPR* false positive rate.

As expected in unbalanced datasets with a much larger number of negative elements than positive ones, NPV was higher than the PPV in all tool-dataset combinations. All NPVs were above 0.96 while PPV varied across datasets, ranging from 0.36 (CoNVaDING in ICR96) to 0.96 (ExomeDepth in In-house MiSeq). ExomeDepth had the highest PPV in all datasets.

Regarding the per gene metric, sensitivity was slightly improved compared to per ROI, and for each dataset, at least one tool showed a sensitivity of 1 and was able to identify all CNVs (Supplementary files 11 and 12).

When excluding single-exon CNVs or multi-exon CNVs, the exclusion of single-exon CNVs generally provided a better PPV, while sensitivity varied depending on the dataset (Supplementary file 13).

### Benchmark with optimized parameters

In addition to evaluating the performance of the different tools tested with default parameters, we performed an optimization process to identify, for each tool and dataset, the combination of parameters that maximized the sensitivity as required for a screening tool in a diagnostics context (see “Methods” and Supplementary files 8 and 9).

**Fig. 3:**
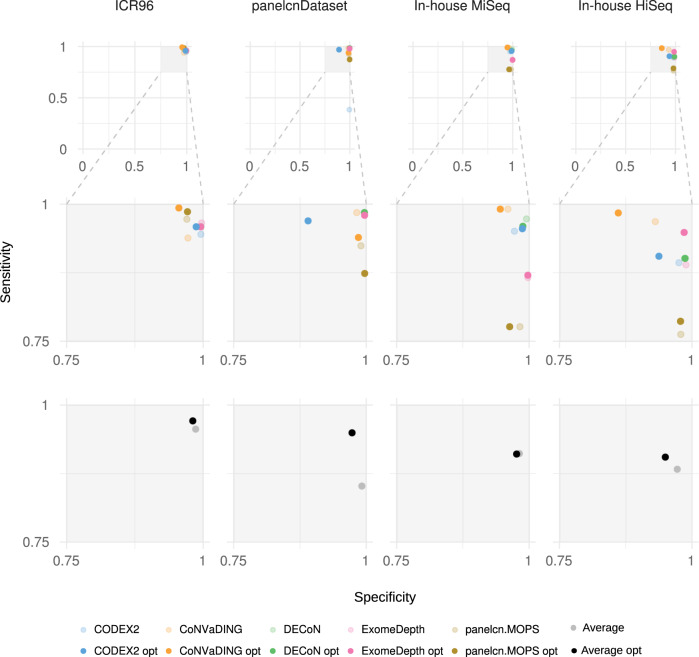
Optimization results at ROI level. Shows sensitivity and specificity on validation sets when executing tools with the optimized parameters in comparison to the default parameters (ROI region of interest).

Parameter optimization was performed on a subset (training) of each dataset and the optimized parameters (Supplementary file 10) were compared to the default ones on the samples not used for training (validation subset). Figure 3 shows the optimization results at the ROI level. In general, the optimization process improved sensitivity by slightly decreasing specificity. For panelcnDataset, sensitivity was increased by a higher margin driven by CODEX2, which increased its sensitivity by 58.6%. On the other hand, tools were not able to improve or showed small differences in the In-house MiSeq dataset (Supplementary files 14 and 15).

### Benchmark in a diagnostics scenario

In a real diagnostic setting, all CNVs detected in genes of interest and all regions where the screening tool was not able to produce a result (no call) should be confirmed by an orthogonal technique. To account for this, we evaluated the performance of all tools using the whole diagnostics strategy metric which takes the no calls into account. This evaluation was performed in a modified version of the in-house datasets, the augmented in-house datasets (Fig. 1), which contained all the samples from the original sequencing runs instead of a selection of them (see “Methods”).

**Fig. 4:**
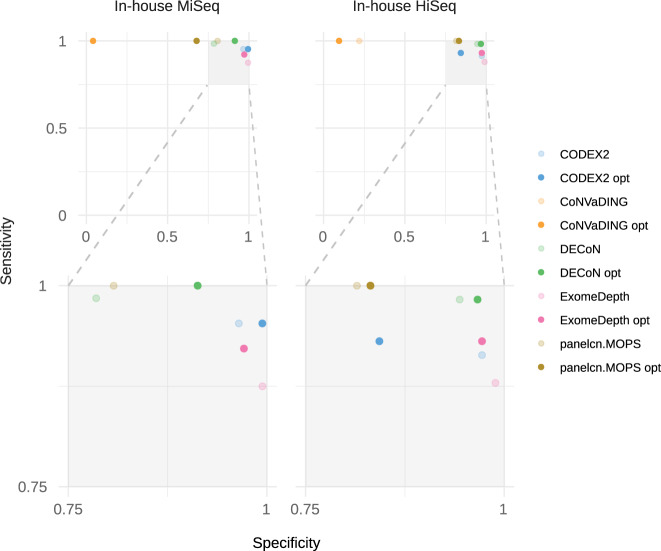
Benchmark results for the diagnostics scenario: whole diagnostics strategy metrics. Shows sensitivity and specificity on the augmented in-house datasets when executing tools with the optimized parameters in comparison to the default parameters.

Figure 4 shows sensitivity and specificity on the augmented in-house datasets when executing tools with the optimized parameters compared to the default parameters. For the In-house MiSeq dataset, two tools detected all CNVs: panelcn.MOPS achieved it with both optimized and default parameters (CI: 94.4–100%), with a specificity of 67.8% (CI: 60.3–74.8%) and 80.7% (CI: 74.0–86.3%), respectively. DECoN detected all CNVs only with the optimized parameters (CI: 94.4–100%) reaching 91.3% (CI: 86.0–95.0%) specificity. CoNVaDING also detected all CNVs, but its high no-call rate led to very low specificity, 4.1% (CI: 1.6–8.2%). For the In-house HiSeq dataset, only panelcn.MOPS detected all CNVs (CI: 93.8–100%) with an acceptable specificity (81.5% (CI: 75.0–86.9%) and 83.2% (CI: 76.8–88.3%) with the default and optimized parameters respectively). DECoN missed one CNV, being a mosaic sample, and its specificity remained high, 96.6% (CI: 92.8–98.8%) with the optimized parameters. On the other hand, CODEX2 and ExomeDepth obtained high sensitivity and specificity values for both datasets, but they did not report no calls (Table 4 and Supplementary files 16 and 17).

**Table 4:** Benchmark results with default and optimized parameters in the diagnostics scenario.

Dataset	Parameters	Tool	TP	TN	FP	FN	Sensitivity	Specificity	F1
In-house MiSeq	Default parameters	DECoN	63	135	37	1	0.9844	0.7849	0.7683
		panelcn.MOPS	64	138	33	0	1	0.807	0.795
		CoNVaDING	64	7	165	0	1	0.0407	0.4369
		exomedepth	56	171	1	8	0.875	0.9942	0.9256
		CODEX2	61	163	6	3	0.9531	0.9645	0.9313
	Optimized parameters	DECoN	64	157	15	0	1	0.9128	0.8951
		panelcn.MOPS	64	116	55	0	1	0.6784	0.6995
		CoNVaDING	64	7	165	0	1	0.0407	0.4369
		exomedepth	59	167	5	5	0.9219	0.9709	0.9219
		CODEX2	61	168	1	3	0.9531	0.9941	0.9683
In-house HiSeq	Default parameters	DECoN	57	168	10	1	0.9828	0.9438	0.912
		panelcn.MOPS	58	145	33	0	1	0.8146	0.7785
		CoNVaDING	58	39	139	0	1	0.2191	0.4549
		exomedepth	51	176	2	7	0.8793	0.9888	0.9189
		CODEX2	53	173	5	5	0.9138	0.9719	0.9138
	Optimized parameters	DECoN	57	172	6	1	0.9828	0.9663	0.9421
		panelcn.MOPS	58	148	30	0	1	0.8315	0.7945
		CoNVaDING	58	17	161	0	1	0.0955	0.4188
		exomedepth	54	173	5	4	0.931	0.9719	0.9231
		CODEX2	54	150	28	4	0.931	0.8427	0.7714

## Discussion

CNVs are the genetic cause of multiple hereditary diseases [2]. To detect them, specific tools and techniques are required. In genetic diagnostics, this is mainly done using either MLPA and aCGH or using software tools to infer copy-number alterations from NGS data generated in the diagnostics process. MLPA and aCGH are the gold standard methods [3], but both are time-consuming and expensive approaches that frequently lead laboratories to only use them in a subset of genes of interest. On the other hand, multiple tools for CNV calling from NGS data have been published [5–7], but their performance on NGS gene panel data has not been properly evaluated in a genetic diagnostics context. This evaluation is especially critical when these tools are used as a screening step in a diagnostics strategy, since a nonoptimal sensitivity would lead to a higher number of misdiagnosis.

Most CNV calling tools have not been developed to be used as a screening step in genetic diagnostics but as part of a research-oriented data analysis pipeline. Therefore, they were originally tuned and optimized for a certain sensitivity-specificity equilibrium. To be used as screening tools, we need to alter their default parameters to shift that equilibrium toward maximizing the sensitivity even at the expense of lowering their specificity. This parameter optimization must be performed in a dataset-specific way, since tools show performance differences between dataset due to dataset specificities coming from target regions composition, technical differences, or sequencing characteristics.

In this work, we selected 5 tools that have shown promising results on panel data, and we measured their performance, with the default and sensitivity-optimized parameters, over 4 validated datasets from different sources: a total of 495 samples with 231 single and multi-exon CNVs. CNVbenchmarkeR, a framework for evaluating CNV calling tools performance, was developed to undertake this task. We also evaluated their performance in a genetic diagnostics-like scenario and showed that some of the tools are suitable to be used as screening methods before MLPA or aCGH confirmation.

### Benchmark with default parameters

The benchmark with default parameters showed that most tools are highly sensitive and specific, but the top performers depend on the specific dataset. Most tools performed best when using data from panelcnDataset. DECoN, ExomeDepth and CoNVaDING reached almost 100% sensitivity and specificity. A possible reason for this is that this dataset contains the lowest number of single-exon CNVs (*n* = 13), which are the most difficult type of CNVs to be detected. DECoN was the best performer for ICR96, a dataset published by the same authors, but other tools obtained similar results in that dataset. CoNVaDING was the most sensitive tool when analyzing our in-house datasets but showed the lowest PPV in all datasets with the exception of panelcnDataset. ExomeDepth showed the highest PPV in all datasets, making it one of the most balanced tools regarding sensitivity and specificity. Differences in tool performance depending on the dataset were also observed in previous works [29, 30].

### Optimization

The different CNV calling tools included in this work were originally designed with different aims with respect to their preferred sensitivity and specificity equilibrium or the type of CNVs they expected to detect, and this is reflected in their default parameters and their performance in the initial benchmark. Our aim with this work was to evaluate these CNV callers as potential screening tools in a genetic diagnostics setting and for this reason, we required their maximum sensitivity.

The parameter optimization process allowed us to determine the dataset-specific parameter combination maximizing their sensitivity without an excessive specificity loss. The optimization had a different impact on different tools: while CODEX2 showed a higher sensitivity in all four datasets the rest of the tools showed modest improvements. This is mainly due to the fact that sensitivity was already over 0.9 for most combinations and the number of false negatives to correctly call was small (between 4 and 8) in the per gene metric.

The final optimized parameters were dataset specific, so we do not recommend using them directly on other datasets where the data have been obtained differently (different capture protocol or sequencing technologies, for example).

Based on our results, we would recommend optimizing the parameters for each specific dataset before adding any CNV calling tool to a genetic diagnostics pipeline to maximize its sensitivity and reduce the risk of misdiagnosis. To that end, we have developed an R framework, CNVbenchmarkeR (freely available at https://github.com/TranslationalBioinformaticsIGTP/CNVbenchmarkeR), that will help to perform the testing and optimization process in any new dataset.

### Diagnostics scenario

Two tools showed performance good enough to be implemented as screening methods in the diagnostics scenario evaluated in our two in-house datasets (Fig. 4): DECoN and panelcn.MOPS. While panelcn.MOPS was able to detect all CNVs both with the default and the optimized parameters, DECoN reached almost perfect sensitivity and outperformed panelcn.MOPS specificity when using the optimized parameters, although the difference is not statistically significant. DECoN only missed a mosaic CNV affecting two exons of the NF2 gene. CoNVaDING also detected all CNVs, but the high number of no-call regions reduced its specificity to values between 4.1 and 21.9%, which rendered it non-valid as a screening tool.

The parameter optimization process improved the sensitivity of most tools. For example, for the In-house MiSeq dataset, DECoN sensitivity increased from 98.4% (CI: 91.6–100%) to 100% (CI: 94.4–100%), and the specificity increased from 78.5% (CI: 71.6–84.4%) to 91.3% (CI: 86.0–95.0%). This improvement highlights the importance of fine-tuning the tool parameters for each specific task, and shows that the optimization process performed in this work has been key for the evaluation of the different tools.

The high sensitivity reached by DECoN and panelcn.MOPS in different datasets, where they identified all known CNVs, shows that NGS data can be used as a CNV screening step in a genetic diagnostics setting. This screening step has the potential to improve the diagnostics routines. As an example, the high specificity reached by DECoN in the in-house MiSeq dataset with the optimized parameters means that around 91% of genes with no CNV would not need to be specifically tested for CNVs when using DECoN as a screening step. The resources saved by the reduction in the number of required tests could be used to expand the number of genes analyzed, potentially increasing the final diagnostics yield.

In conclusion, according to our analysis, DECoN and panelcn.MOPS provide the highest performance for CNV screening before orthogonal confirmation. Although panelcn.MOPS showed a slightly higher sensitivity in one of the datasets, DECoN showed a much higher specificity in the diagnostics scenario. Our results also showed that tools performance depends on the dataset. Therefore, it may be important to evaluate potential tools on an in-house dataset before implementing one as a screening method in the diagnostics routine.

## Supplementary information

Supplementary Files Legends

Supplementary File 1

Supplementary File 2

Supplementary File 3

Supplementary File 4

Supplementary File 5

Supplementary File 6

Supplementary File 7

Supplementary File 8

Supplementary File 9

Supplementary File 10

Supplementary File 11

Supplementary File 12

Supplementary File 13

Supplementary File 14

Supplementary File 15

Supplementary File 16

Supplementary File 17

## References

[1] Teo SM, Pawitan Y, Ku CS, Chia KS, Salim A (2012). Statistical challenges associated with detecting copy number variations with next-generation sequencing. Bioinformatics.

[2] Zhang F, Gu W, Hurles ME, Lupski JR (2009). Copy number variation in human health, disease, and evolution. Annu Rev Genomics Hum Genet.

[3] Kerkhof J, Schenkel LC, Reilly J, McRobbie S, Aref-Eshghi E, Stuart A (2017). Clinical validation of copy number variant detection from targeted next-generation sequencing panels. J Mol Diagn.

[4] Talevich E, Shain AH, Botton T, Bastian BC (2016). CNVkit: genome-wide copy number detection and visualization from targeted DNA sequencing. PLoS Comput Biol.

[5] Zhao M, Wang Q, Wang Q, Jia P, Zhao Z (2013). Computational tools for copy number variation (CNV) detection using next-generation sequencing data: features and perspectives. BMC Bioinforma.

[6] Abel HJ, Duncavage EJ (2013). Detection of structural DNA variation from next generation sequencing data: a review of informatic approaches. Cancer Genet.

[7] Mason-Suares H, Landry L, S. Lebo M (2016). Detecting copy number variation via next generation technology. Curr Genet Med Rep.

[8] Truty R, Paul J, Kennemer M, Lincoln SE, Olivares E, Nussbaum RL (2019). Prevalence and properties of intragenic copy-number variation in Mendelian disease genes. Genet Med.

[9] Johansson LF, van Dijk F, de Boer EN, van Dijk-Bos KK, Jongbloed JDH, van der Hout AH (2016). CoNVaDING: Single Exon Variation Detection in Targeted NGS Data. Hum Mutat.

[10] Fowler A, Mahamdallie S, Ruark E, Seal S, Ramsay E, Clarke M (2016). Accurate clinical detection of exon copy number variants in a targeted NGS panel using DECoN. Wellcome Open Res.

[11] Povysil G, Tzika A, Vogt J, Haunschmid V, Messiaen L, Zschocke J (2017). panelcn.MOPS: Copy number detection in targeted NGS panel data for clinical diagnostics. Hum Mutat.

[12] Kim H-Y, Choi J-W, Lee J-Y, Kong G, Kim H-Y, Choi J-W (2017). Gene-based comparative analysis of tools for estimating copy number alterations using whole-exome sequencing data. Oncotarget.

[13] Chiang T, Liu X, Wu TJ, Hu H, Sedlazeck FJ, White S (2019). Atlas-CNV: a validated approach to call single-exon CNVs in the eMERGESeq gene panel. Genet Med.

[14] Roca I, González-Castro L, Fernández H, Couce ML, Fernández-Marmiesse A (2019). Free-access copy-number variant detection tools for targeted next-generation sequencing data. Mutat Res/Rev Mutat Res.

[15] Mahamdallie S, Ruark E, Yost S, Ramsay E, Uddin I, Wylie H (2017). The ICR96 exon CNV validation series: a resource for orthogonal assessment of exon CNV calling in NGS data. Wellcome Open Res.

[16] Castellanos E, Gel B, Rosas I, Tornero E, Santín S, Pluvinet R (2017). A comprehensive custom panel design for routine hereditary cancer testing: Preserving control, improving diagnostics and revealing a complex variation landscape. Sci Rep.

[17] Plagnol V, Curtis J, Epstein M, Mok KY, Stebbings E, Grigoriadou S (2012). A robust model for read count data in exome sequencing experiments and implications for copy number variant calling. Bioinformatics.

[18] Jiang Y, Wang R, Urrutia E, Anastopoulos IN, Nathanson KL, Zhang NR (2018). CODEX2: Full-spectrum copy number variation detection by high-throughput DNA sequencing. Genome Biol.

[19] Li H, Durbin R (2009). Fast and accurate short read alignment with Burrows-Wheeler transform. Bioinformatics.

[20] Li H (2013). Aligning sequence reads, clone sequences and assembly contigs with BWA-MEM. arXiv.

[21] Li H, Handsaker B, Wysoker A, Fennell T, Ruan J, Homer N (2009). The sequence alignment/map format and SAMtools. Bioinformatics.

[22] Flicek P, Amode MR, Barrell D, Beal K, Brent S, Carvalho-Silva D (2012). Ensembl 2012. Nucleic Acids Res.

[23] Gentleman R, Carey V, Bates D, Bolstad B, Dettling M, Dudoit S (2004). Bioconductor: open software development for computational biology and bioinformatics. Genome Biol.

[24] Wickham H (2011). The split-apply-combine strategy for data analysis. J Stat Softw.

[25] Lawrence M, Huber W, Pagès H, Aboyoun P, Carlson M, Gentleman R (2013). Software for computing and annotating genomic ranges. PLoS Comput Biol.

[26] Durinck S, Spellman PT, Birney E, Huber W (2009). Mapping identifiers for the integration of genomic datasets with the R/bioconductor package biomaRt. Nat Protoc.

[27] Wickham H. ggplot2: elegant graphics for data analysis. New York: Springer-Verlag; 2016. 10.18637/jss.v077.b02.

[28] de Ligt J, Boone PM, Pfundt R, Vissers LELM, Richmond T, Geoghegan J (2013). Detection of clinically relevant copy number variants with whole exome sequencing. Hum Mutat.

[29] Sadedin SP, Ellis JA, Masters SL, Oshlack A (2018). Ximmer: a system for improving accuracy and consistency of CNV calling from exome data. Gigascience.

[30] Hong CS, Singh LN, Mullikin JC, Biesecker LG (2016). Assessing the reproducibility of exome copy number variations predictions. Genome Med.

