# Supplementary material for: Evaluation of CNV detection tools for NGS panel data in genetic diagnostics
Source: Eur J Hum Genet. 2020 Jun 19;28(12):1645–55. doi: 10.1038/s41431-020-0675-z (PMC7784926; doi:10.1038/s41431-020-0675-z)
Supplement: Supplementary file 1 — Supplementary Files Legends [file 41431_2020_675_MOESM1_ESM.docx]

**Supp File 1** (pdf) *Explanation of IBK141 sample exclusion from EGAD00001003335 dataset (panelcnDataset).*

**Supp File 2** (xlsx) *MLPA-detected CNVs for ICR96 dataset.*

**Supp File 3** (xlsx) *MLPA-detected CNVs for panelcnDataset dataset. (Indication: set of genes tested by MLPA; ruth: MLPA result [CN0: homozygous deletion; CN1: heterozygous deletion; CN2: normal; CN3: duplication (1 copy); CN4: duplication (2 copies)].*

**Supp File 4** (xlsx) *MLPA-detected CNVs for in-house MiSeq dataset. (Indication: set of genes tested by MLPA; Truth: MLPA result [CN0: homozygous deletion; CN1: heterozygous deletion; CN2: normal; CN3: duplication (1 copy); CN4: duplication (2 copies)].*

**Supp File 5** (xlsx) *MLPA-detected CNVs for in-house HiSeq dataset. (Indication: set of genes tested by MLPA; Truth: MLPA result [CN0: homozygous deletion; CN1: heterozygous deletion; CN2: normal; CN3: duplication (1 copy); CN4: duplication (2 copies)].*

**Supp File 6** (xlsx) *Targets bed file used for ICR96 and panelcnDataset datasets.*

**Supp File 7** (xlsx) *Targets bed file used for in-house datasets.*

**Supp File 8** (xlsx) *Training and validation sets for all datasets.*

**Supp File 9** (docx) *Optimization algorithm description, pseudocde and parameters.*

**Supp File 10** (xlsx) *Default and sensitivity-optimized parameters for all tools and datasets.*

**Supp File 11** (eps) *Benchmark results with default parameters: per gene metrics. Shows results when executing tools with the default parameters and computing the per gene metrics. (PPV: positive predictive value; F1: F1 score)*

**Supp File 12** (xlsx) *Benchmark results when evaluating tools with the default parameters.*

**Supp File 13** (xlsx) *Benchmark results when evaluating tools with the default parameters dividing the datasets into single-exon and multi-exon.*

**Supp File 14** (xlsx) *Benchmark results when evaluating tools with the default and sensitivity-optimized parameters against the validation subsets of each dataset.*

**Supp File 15** (xlsx) *Benchmark results when evaluating tools with the default and sensitivity-optimized parameters against the validation subsets diving them into single-exon and multi-exon.*

**Supp File 16** (xlsx) *Benchmark results for the diagnostics scenario: metrics on the augmented in-house datasets when executing tools with the optimized parameters in comparison to the default parameters on the validation subsets.*

**Supp File 17** (xlsx) *Benchmark results for the diagnostics scenario: metrics on the augmented in-house datasets when executing tools with the optimized parameters in comparison to the default parameters on the validation subsets divinding the datasets into single-exon and multi-exon.*
